# Prognostic value of microRNAs in patients with small cell lung cancer: a meta-analysis

**DOI:** 10.1186/s12957-022-02851-4

**Published:** 2022-12-04

**Authors:** Jun Peng, Jinfeng Liu, Huining Liu, Yan Huang, Yingchun Ren

**Affiliations:** 1 Department of Thoracic Surgery, The First Hospital of Hebei Medical University, No. 89, Donggang Road, Yuhua District, Shijiazhuang City, Hebei Province China; 2 Clinical Medical College of Jining Medical College, No. 45, Jianshe South Road, Rencheng District, Jining City, Shandong Province China

**Keywords:** Meta-analysis, MicroRNAs, Small cell lung cancer, Prognosis

## Abstract

**Background:**

An increasing number of studies have shown that microRNAs play an important role in the occurrence and development of small cell lung cancer, which mainly manifest as oncogenic and tumor inhibition. Therefore, microRNAs may affect the survival of patients with small cell lung cancer. In this meta-analysis, we will evaluate the role of microRNAs in the overall survival of patients with small cell lung cancer, which may provide valuable information for the treatment of small cell lung cancer.

**Methods:**

We searched the PubMed, Embase, and Web of Science online databases to determine the effect of microRNAs on the prognosis of patients with small cell lung cancer. The data and characteristics of each study were extracted, and the hazard ratios (HRs) and 95% confidence intervals (CIs) were calculated to estimate the effect.

**Results:**

A total of 7 articles, involving 427 subjects and 15 studies, were included in this meta-analysis. The pooled HR of the relationship between the microRNA expression level and the overall survival rate of small cell lung cancer patients was 1.25 (95% CI: 1.06–1.47). There was a significant difference in the prognostic value of oncogenic and tumor inhibition microRNAs among patients with small cell lung cancer, with pooled HRs of 1.60 (95% CI: 1.35–1.90) and 0.42 (95% CI: 0.30–0.57), respectively.

**Conclusions:**

MicroRNAs have a significant impact on the overall survival of small cell lung cancer patients, suggesting that microRNAs can be used as potential prognostic markers and may provide treatment strategies for small cell lung cancer patients.

**Trial registration:**

The protocol was registered on PROSPERO website with the registration number of CRD42022334363. The relevant registration information can be obtained from the website https://www.crd.york.ac.uk/prospero/#searchadvanced.

**Supplementary Information:**

The online version contains supplementary material available at 10.1186/s12957-022-02851-4.

## Background

Lung cancer has the highest mortality in the world [1] and can be categorized into non-small-cell lung cancer (NSCLC) and small cell lung cancer (SCLC). SCLC is a highly aggressive disease, accounting for approximately 13–15% of all lung cancers [2]. In recent decades, the main treatment for SCLC has been chemotherapy [3], and the main first-line chemotherapy regimen is the combination of etoposide and cisplatin [4]. The initial stage of SCLC treatment is sensitive to chemotherapy, but it soon develops into a drug-resistance stage, and the deterioration of the tumor is accelerated [5].

In recent years, an increasing number of reports have shown that miRNAs can affect the prognosis of SCLC [6–12]. MiRNAs are small noncoding RNAs containing 20 to 23 nucleotide molecules. They are usually combined with complementary sequences in the 3′ untranslated region (3′UTR) of target genes to influence gene expression [13], which plays a role in biological processes such as cell proliferation and cycle regulation, cell apoptosis, cell invasion, autophagy, and cell DNA repair [14–16]. Dysfunction of miRNAs is a common event in many human tumors and disturbs the expression of oncogenic or tumor-suppressive target genes [17], which plays an important role in the occurrence and development of tumors, such as increasing the invasiveness in breast cancer [18], participating in the development of leukemia [19], and participating in the occurrence and development of lung cancer [20]. The clinical utility of miRNA expression analysis in predicting the efficacy of treatment strategies, including surgery, chemoradiotherapy, and targeted therapy, has been evaluated in small cell lung cancer [21]. This meta-analysis aims to analyze relevant studies to produce reliable results regarding whether miRNAs can be used as reliable prognostic biomarkers in patients with small cell lung cancer.

## Methods

### Search strategy

The PubMed, Embase, and Web of Science online databases were searched for relevant studies by three reviewers, and the following keyword combinations were used to retrieve relevant studies: (“small cell lung cancer” or “SCLC”) and (“microRNAs” or “microRNA” or “miRNAs” or “miRNA” or “miR”) and (“survival” or “prognosis” or “mortality”). Including articles published in all languages, studies carried out on human subjects and articles published from the establishment of the database to March 1, 2022. The search strategies were adapted according to the characteristics of the databases (see Supplementary Table 1 for more details). The protocol was registered on PROSPERO website with the registration number of CRD42022334363. The relevant registration information can be obtained from the website: https://www.crd.york.ac.uk/prospero/#searchadvanced. The three authors manually screened the reference lists of the included articles to identify additional relevant studies.

If two or more studies with different results were carried out in the same article and the HR of the 95% CI or the corresponding results could be calculated by Kaplan–Meier curve, we considered them to be separate publications. When univariate and multivariate analyses were performed simultaneously, we chose the latter as a more accurate result. In addition, when different publications investigated patients in the same cohort, we selected the most complete study.

### Inclusion and exclusion criteria

The inclusion criteria were as follows: (1) patients with SCLC were studied and (2) there was a relationship between miRNA expression and the overall survival rate of patients with SCLC. The exclusion criteria were as follows: (1) tissues or materials from animals other than humans were studied; (2) the research focused on other types of cancer rather than just SCLC; (3) the survival results were not reported or could not be calculated; and (4) overviews, reviews, seminar papers, comments, reports, letters, and duplicate publications were excluded.

### Data extraction and quality assessment

The following data were extracted from all included papers by two independent reviewers: the first author’s name, publication year, country and region, number of subjects, sample source, miRNA type, patient treatment, follow-up time (basic unit: month), HR, 95% CI, and overall survival (OS) and progression-free survival (PFS) (Table 1). If a study did not provide the HR and 95% CI, we extracted these data using Engauge Digitizer11.1. After calculation, we obtained the HR of 8 studies. We collected all HRs to determine high and low expression levels of miRNA. Follow-up time was determined by reading the original article or by examining Kaplan–Meier curves. Disagreements were resolved by discussion among the two investigators and by consulting with a third investigator.

**Table 1:** The basic characteristics of included studies

Study ID	miRNA type	Prognosis	Resources	Follow-up time	miRNA panel	Influence	NOS	Region	Year
Mancuso et al. [6]	miR-192	OS	Tumor	83 months	1	Oncogenic	8	Italy	2016
	miR-200c	OS	Tumor	83 months	1	Oncogenic	8		
	3-miRNA	OS	Tumor	83 months	3	Oncogenic	8		
Cao et al. [7]	miR-886-3p	OS	Tumor	120 months	1	Tumor suppressive	8	China	2013
	miR-886-3p	PFS	Tumor	120 months	1	Tumor suppressive	8		
Zhou et al. [8]	miR-184	PFS	Serum	27 months	1	Tumor suppressive	7	China	2015
	miR-574-5p	PFS	Serum	27 months	1	Oncogenic	7		
	miR-574-5p	OS	Serum	27 months	1	Oncogenic	7		
Liu et al. [9]	miRNA-7	OS	Tumor	50 months	1	Tumor suppressive	8	China	2015
Li et al. [10]	miRNA-92b	PFS	Plasma	30 months	1	Oncogenic	6	China	2020
	miR-375	PFS	Plasma	30 months	1	Oncogenic	6		
	2-miRNA	PFS	Plasma	30 months	2	Oncogenic	6		
	2-miRNA	OS	Tumor	120 months	2	Tumor suppressive	9		
Bi et al. [11]	2-miRNA	PFS	Tumor	120 months	2	Tumor suppressive	9	China	2014
Ranade et al. [12]	miR-92a-2	OS	Tumor	80 months	1	Oncogenic	8	America	2010

*OS* overall survival, *PFS* progression-free survival

Two reviewers independently evaluated the quality of the selected studies using the Newcastle–Ottawa quality assessment scale (NOS), and the scores of each study ranged from 0 to 9. The scoring method includes three parts: selection (0–4 points), comparability (0–2 points), and result evaluation (0–3 points). Studies with NOS scores ≥ 6 were considered to be of high quality.

### Statistical analysis

Stata 14.0 was used for the meta-analysis. For studies that did not report HRs, Kaplan–Meier curves were obtained from the original literature. Using the Engauge Digitizer11.1 software, 33 points were selected in each graph, and 33 corresponding *X* and *Y* values were obtained for calculation. We calculated the HR and 95% CI for each publication three times independently and chose the average as the final value for analysis. According to the obtained HRs and 95% CIs, patients with poor prognosis of SCLC often have overexpressed miRNA, and the HR is greater than 1. Heterogeneity was assessed by Cochran’s *Q*-test and Higgins’s *I*^2^ statistics. When *I*^2^ < 50% and *P* < 0.10, heterogeneity was considered to be low, and the fixed effects model was used. When *I*^2^ > 50% and *P* < 0.10, heterogeneity was considered to be high, and the random effects model was used. We conducted meta-analyses mainly based on the effects of carcinogenic and suppressive miRNAs, patient prognostic indicators, miRNA combinations, and miRNAs from different sources in vivo. Publication bias was visualized by a funnel plot analyzed by Egger’s and Begg’s bias tests.

## Results

### Characteristics of eligible studies and quality assessment

**Fig. 1:**
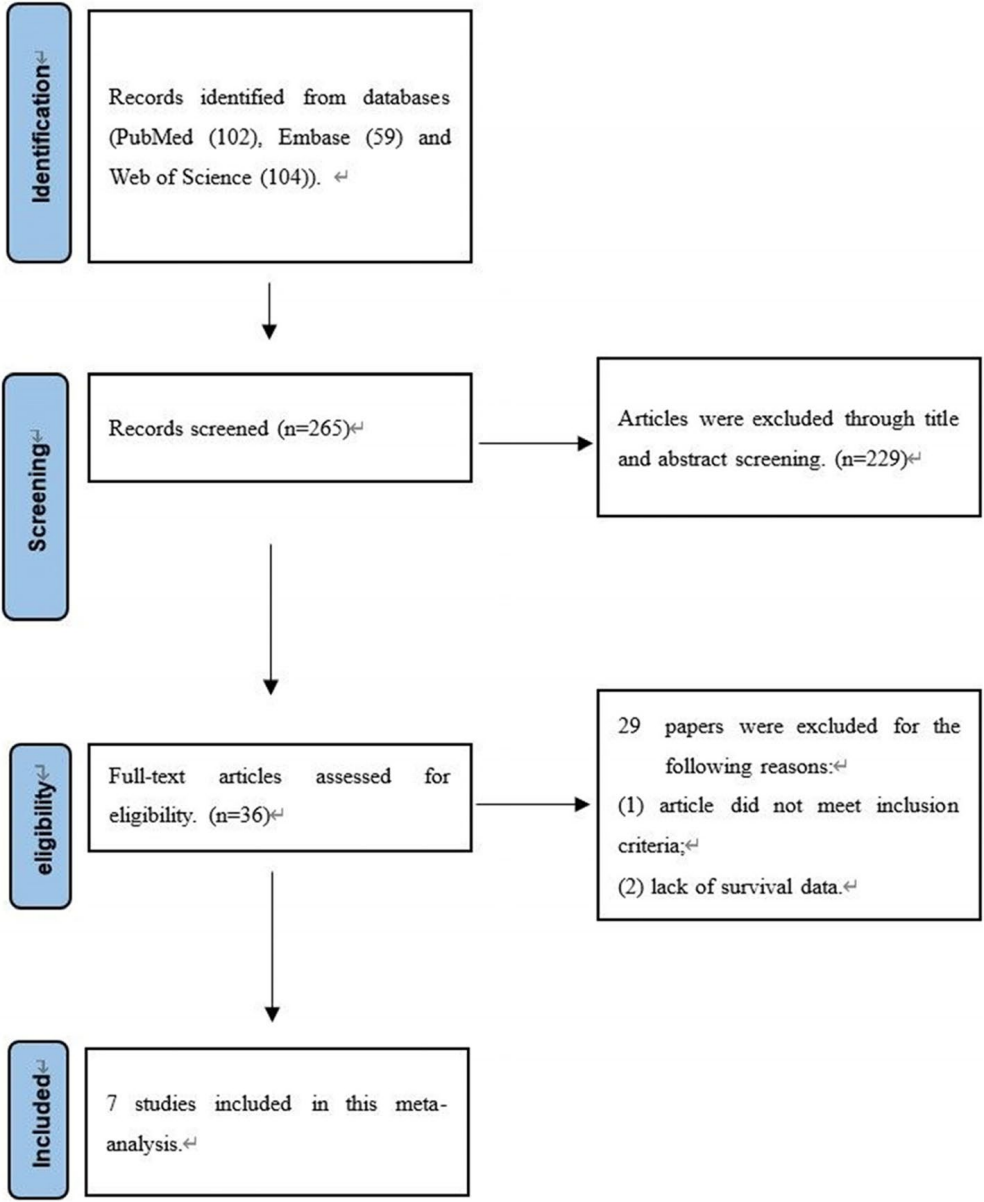
The flow diagram for retrieving eligible articles

Through a systematic literature review, 265 papers were retrieved. After screening the titles and abstracts, 229 articles were excluded due to being irrelevant. Next, we conducted a full-text review of the remaining 36 articles, and 7 studies were determined to be eligible for the current meta-analysis. The flow chart of the research selection process is shown in Fig. 1. The results of the subgroup analysis are shown in Table 2. All of the studies used quantitative reverse transcription polymerase chain reaction (QRT-PCR). We summarized the main characteristics of the seven included studies, and all of them were of high quality (Table 1).

**Table 2:** Results of the subgroup analysis

Subgroup	HR	LL	UL	*N*	*I*^2^	*P*	Heterogeneity between groups: *p* value
Total	1.25	1.06	1.47	15	89.7%	0.000	
Influence							0.000
Oncogenic	1.60	1.35	1.60	9	92.1%	0.000	
Tumor suppressive	0.42	0.30	0.57	6	0.0%	0.501	
Prognosis							0.778
OS	1.20	1.02	1.42	8	89.8%	0.000	
PFS	1.09	0.53	2.22	7	90.5%	0.000	
Resources							0.000
serum	2.15	0.57	8.17	3	95.6%	0.000	
plasma	1.98	1.41	2.80	3	0.0%	0.551	
tumor	0.97	0.84	1.12	9	84.2%	0.000	
miRNA Panel							0.215
1	1.25	1.06	1.48	11	90.9%	0.000	
2	0.64	0.14	3.00	3	90.8%	0.000	
3	2.10	1.10	4.01	1	0.0%	.	
Follow-up time							0.056
<50 months	1.84	0.97	3.46	8	87.8%	0.000	
>50 months	0.97	0.84	1.1290.8$	7	86.1%	0.000	

*LL* lower limit, *UL* upper limit

### Meta-analysis

**Fig. 2:**
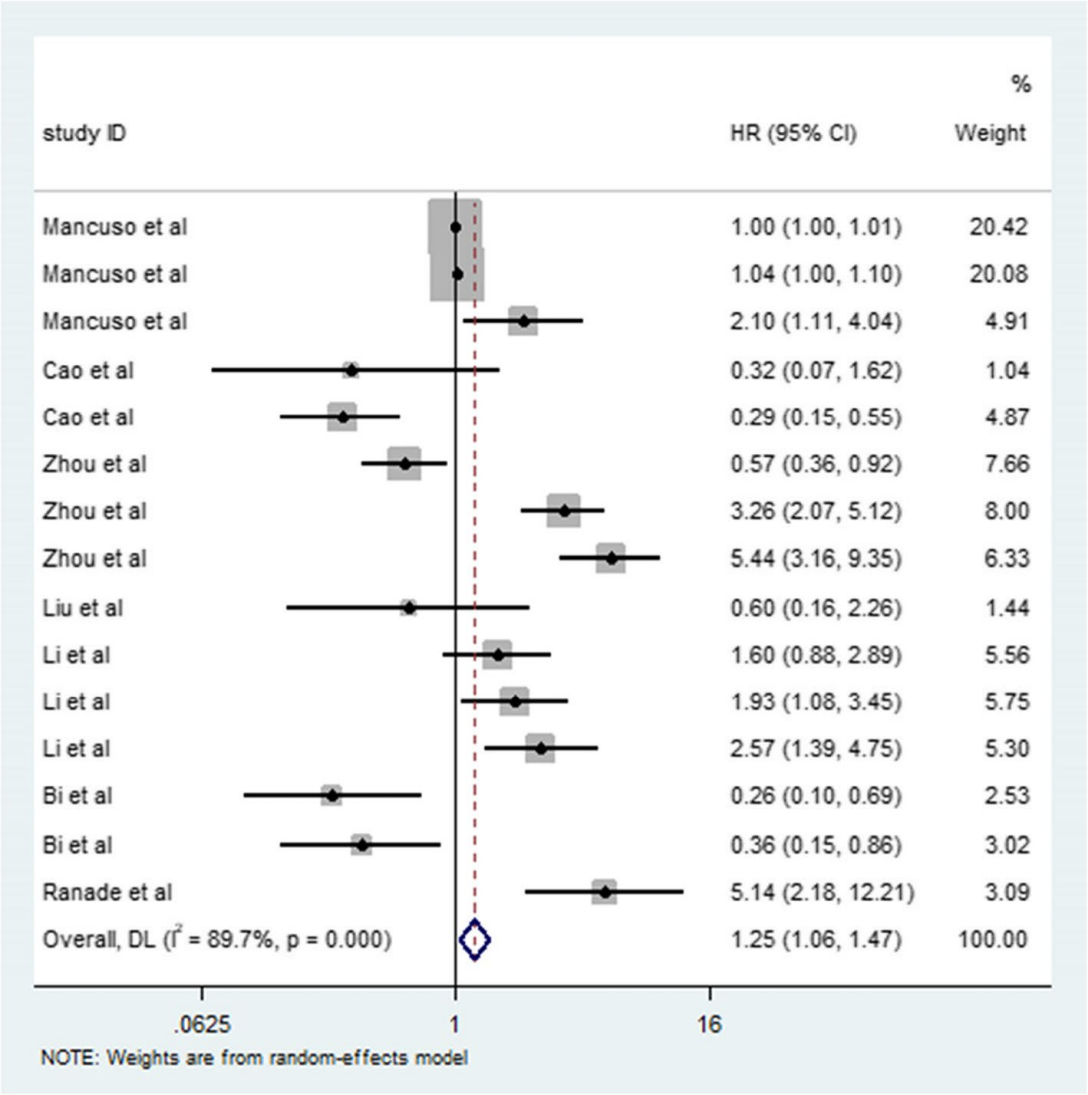
Meta-analysis of total HRs based on microRNAs

The effect of miRNAs on the prognosis of patients with SCLC was studied by meta-analysis. Oncogenic miRNAs and tumor suppressor miRNAs were analyzed to obtain their HRs (Fig. 2), which were 1.60 (95% CI: 1.35–1.90, *I*^2^ = 92.1%, *P* = 0.000) and 0.42 (95% CI: 0.30–0.57, *I*^2^ = 0.0%, *P* = 0.501), respectively. We found that the panel of miRNA types has different effects on the prognosis of patients. The HR of a single miRNA panel was 1.25 (95% CI: 1.06–1.48); the HR of two miRNA panels was 0.64 (95% CI: 0.14–3.00); and the HR of three miRNA panels was 2.10 (95% CI: 1.10–4.01). In addition, the HRs of miRNA on OS and PFS were 1.20 (95% CI: 1.02–1.42, *I*^2^ = 89.8%, *P* = 0.000) and 1.09 (95% CI: 0.53–2.22, *I*^2^ = 90.5%, *P* = 0.000), respectively.

### Relationship between oncogenic miRNAs and tumor suppressor miRNAs and the prognosis of SCLC patients

The correlation between the miRNA found and its role as either oncogenic or suppressive is as follows: MiR-192, miR-200c, miR-574-5p, miR-92b-3p, and miR-375 affect the RB1 gene [22], tumor suppressor gene PTEN [23], tumor suppressor gene 1 (CHES1) [24], Fbxw7 and homeobox D10 [25, 26], SEC23A and YAP1 and YBX1 [27, 28], leading to the occurrence and development of tumors. However, high levels of miR-92a-2 * can lead to chemoresistance in SCLC patients. MiR-886 affects PKR and its downstream pathway, eIF2α phosphorylation, and NF-κB [29]. MiR-7 and miR-184 directly target Bcl-2, c-my, and Bcl-2 [30, 31], leading to tumor suppression.

**Fig. 3:**
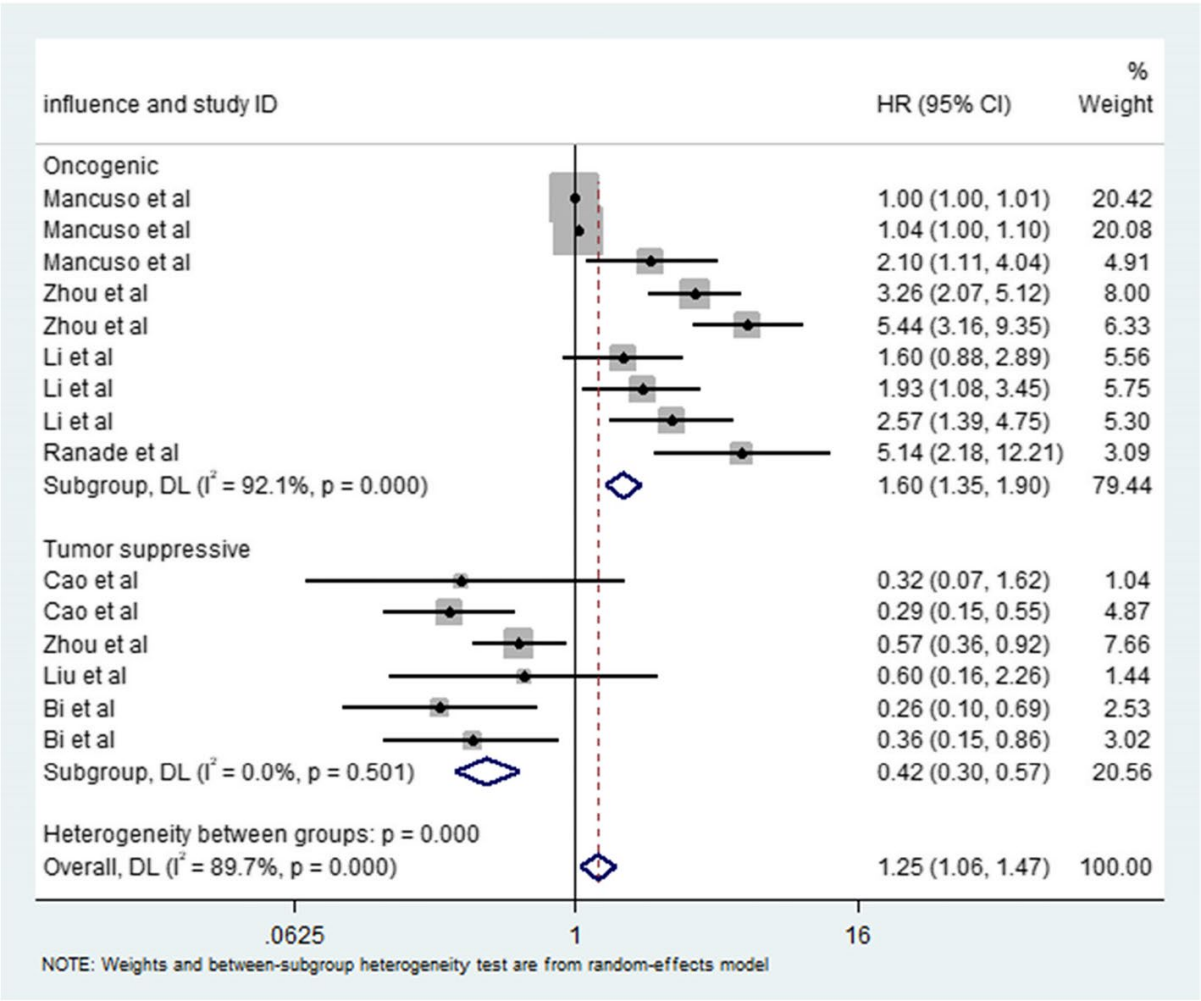
Meta-analysis of subtotal HRs based on different functions in SCLC patients

In our meta-analysis, we compared the relationship between oncogenic miRNAs and tumor suppressor miRNAs and the prognosis of SCLC patients. The results showed that oncogenic miRNAs and tumor suppressor miRNAs were significantly correlated with prognosis, and the pooled HRs were 1.60 (95% CI: 1.35–1.90) (Fig. 3) and 0.42 (95% CI: 0.30–0.57) (Fig. 3), respectively. Therefore, the evidence shows that an increase in tumor suppressive microRNA expression and a decrease in oncogenic microRNA expression are conducive to the prognosis of advanced SCLC, which may indicate that miRNAs can be used as biomarkers to predict the prognosis of patients with small cell lung cancer.

### Relationship between different miRNA panels and the prognosis of patients with small cell lung cancer

**Fig. 4:**
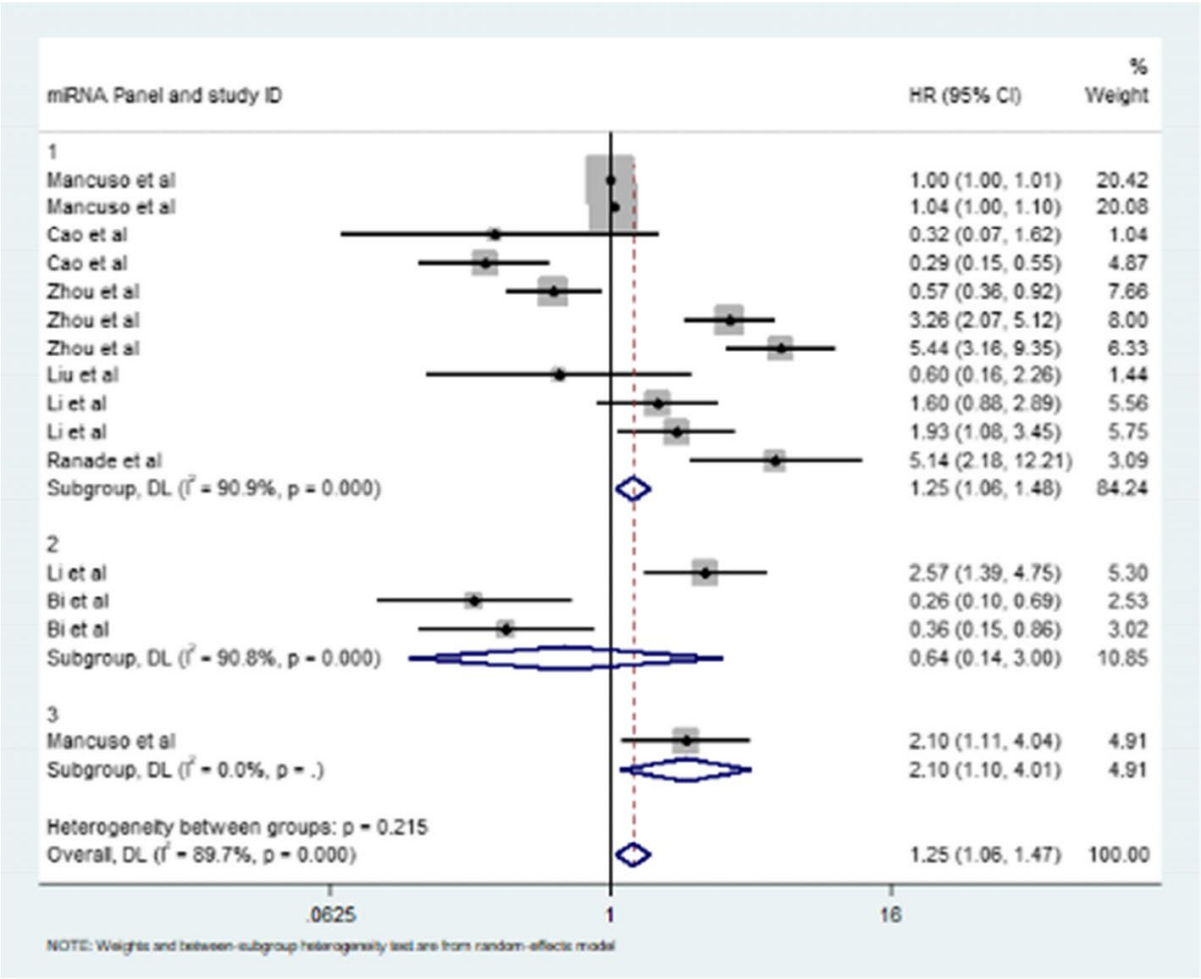
Meta-analysis of subtotal HRs based on different miRNA panels

In our meta-analysis, we further compared the interaction between different miRNA panels and the prognosis of patients with SCLC. Our results clearly showed that there was a significant correlation between a single miRNA panel and the prognosis of patients with SCLC, and the pooled value of HR was 1.25 (95% CI: 1.06–1.48) (Fig. 4); there was also no significant correlation between the presence of two miRNA panels and the prognosis of patients with SCLC, and the pooled value of HR was 0.64 (95% CI: 0.14–3.00) (Fig. 4). When the three miRNA panels were combined, the pooled value of HR was 2.10 (95% CI: 1.10–4.01) (Fig. 4). Therefore, these results may indicate that the presence of a single miRNA effect or three miRNA panels is significantly correlated with the prognosis of patients with SCLC, while the presence of two miRNA panels is not significantly correlated with the prognosis of patients with SCLC.

### Effect of miRNA on prognosis, OS, and PFS in patients with small cell lung cancer

**Fig. 5:**
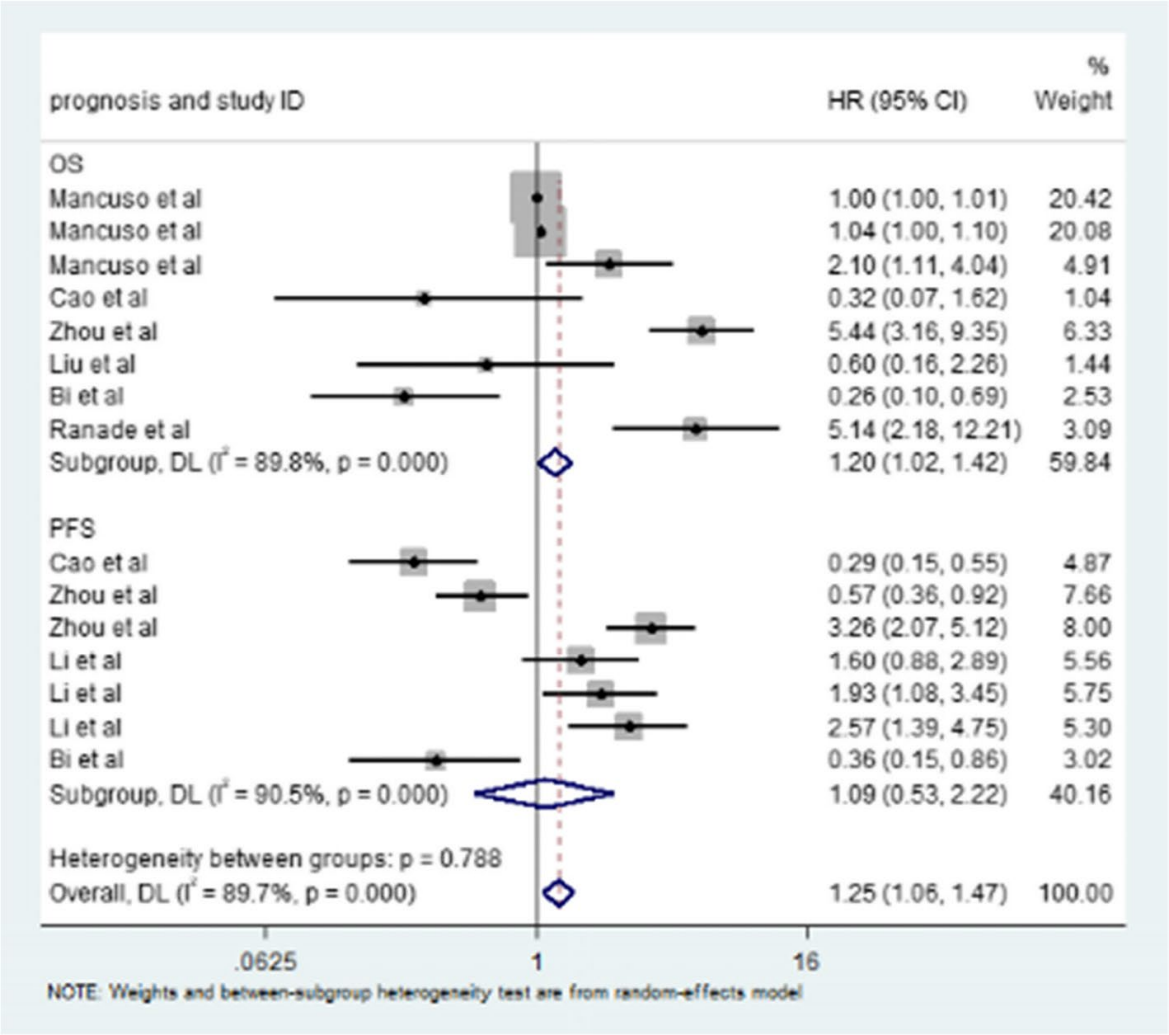
Meta-analysis of subtotal HRs based on the OS and PFS of SCLC patients

In our meta-analysis, we compared the relationship between miRNAs and OS and PFS. Our results clearly showed that there was a significant correlation between miRNA and OS, and the pooled HR was 1.20 (95% CI: 1.02–1.42; Fig. 5). There was no significant correlation between miRNA and PFS, and the pooled HR was 1.09 (95% CI: 0.53–2.22; Fig. 5). Therefore, miRNAs can be used as biomarkers for predicting OS in SCLC patients, but miRNAs may not be used as biomarkers for predicting PFS in SCLC patients. This may be due to the short progression, high degree of malignancy, rapid progression of SCLC after survival, and different miRNAs have different effects, which highlights the value of our study. This effect has been observed in previous studies.

### Sensitivity analysis and publication bias

**Fig. 6:**
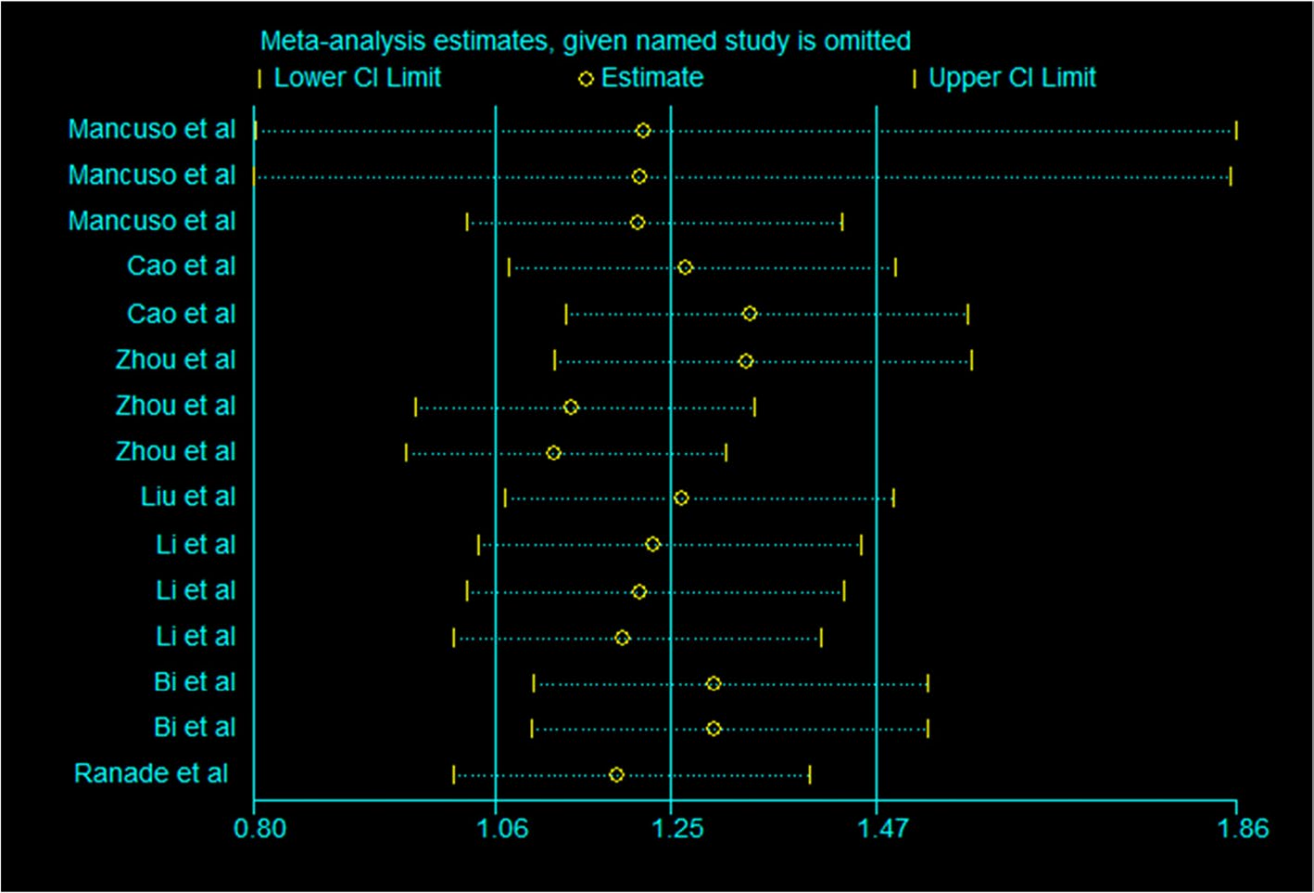
Sensitivity analysis plot

**Fig 7.**
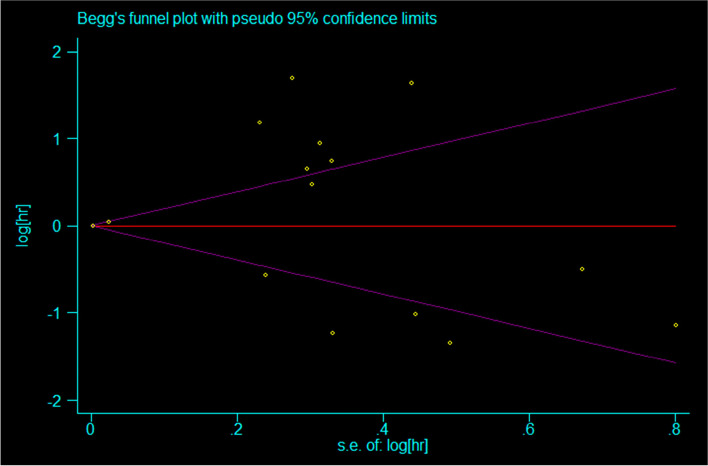
Begg’s funnel plot

**Fig. 8:**
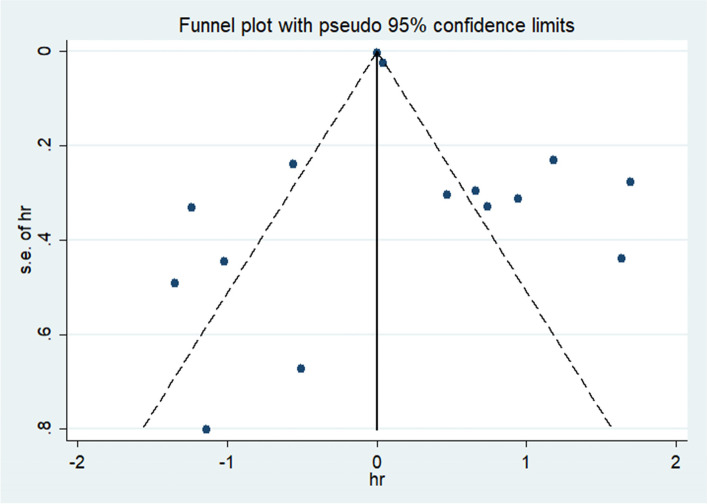
The funnel plot

In the overall meta-analysis, there was significant heterogeneity. Therefore, sensitivity analysis was carried out to explore the source of heterogeneity (Fig. 6). The results were similar regardless of whether the fixed effects model or the random effects model was applied. We used Begg’s funnel chart (Fig. 7) and Egger’s linear regression test to examine publication bias. The Egger test showed that there was no significant publication bias in this study (*P* > 0.05). The shape of the funnel diagram was visually symmetrical, and there was no evidence of publication bias (Fig. 8).

## Discussion

At present, microRNAs are widely studied biomarkers all over the world [32]. We summarized the research progress of microRNAs in SCLC in recent years and focused on the application of microRNAs in the diagnosis, prognosis, and treatment of SCLC.

**Fig. 9:**
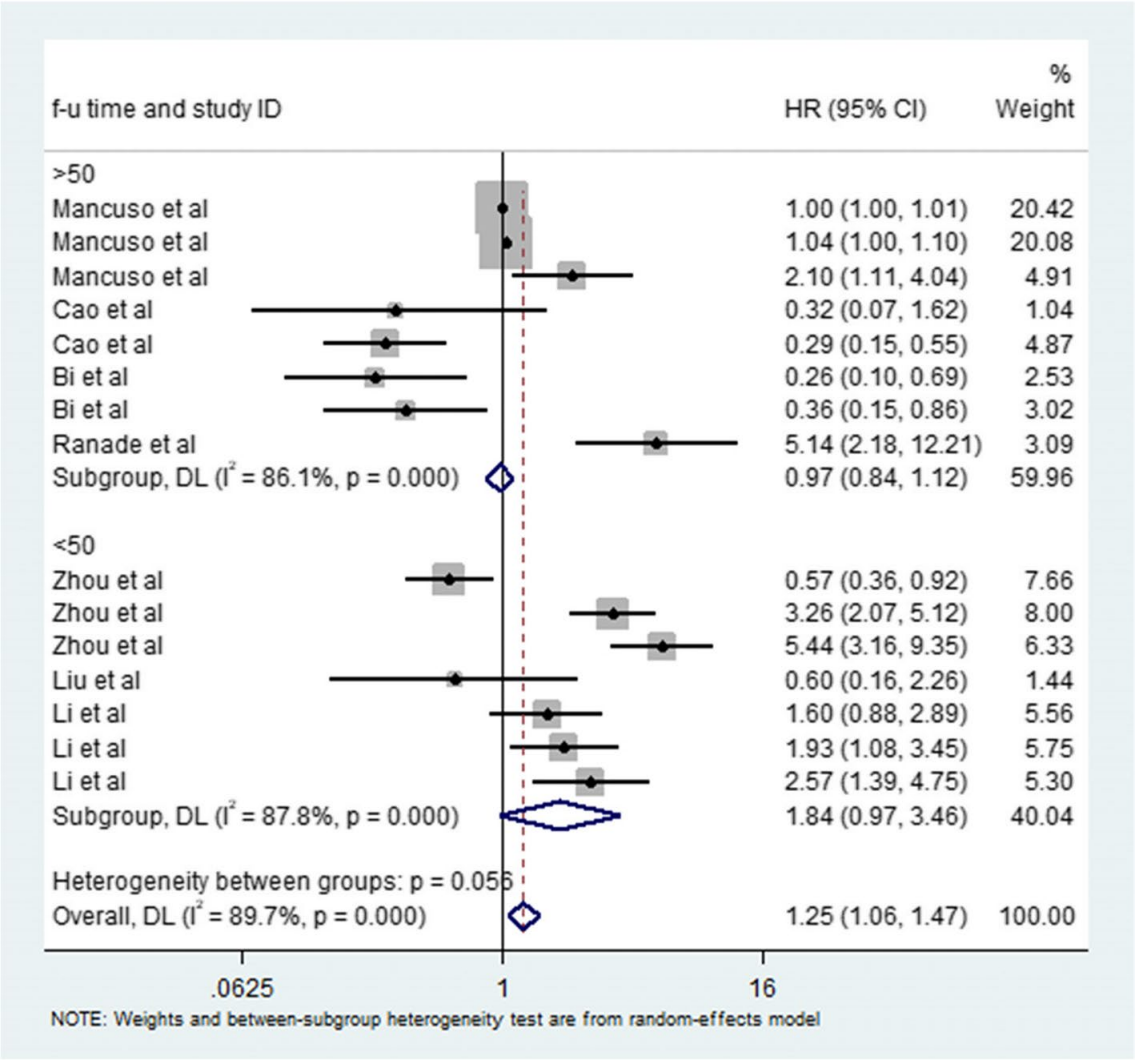
Meta-analysis of subtotal HRs based on follow-up time

In this meta-analysis, we found that high microRNA expression levels were associated with poor prognosis in patients with SCLC with HRs greater than 1. We observed significant heterogeneity in this study and explored it by sensitivity analysis. We found that no specific study affected the overall HR. Since microRNAs play different roles in the occurrence and development of SCLC, we performed subgroup analysis based on oncogenic microRNAs and tumor suppressor microRNAs. They showed different HRs and different levels of heterogeneity, which may explain the source of heterogeneity in the overall meta-analysis. The HR of tumor suppressor microRNAs was significantly lower than that of oncogenic microRNAs, suggesting that SCLC patients with high expression of tumor suppressor microRNAs and low expression of oncogenic microRNAs have a better prognosis. We also performed subgroup analysis of OS and PFS in patients with SCLC. The results showed that microRNAs could be used as biomarkers for OS prediction in SCLC patients. In addition, because the specific miRNAs in different studies are different, different oncogenic miRNAs have different effects on prognosis, and different tumor suppressor miRNAs have different effects on prognosis. Therefore, when two or more oncogenic miRNAs and tumor suppressor miRNAs act simultaneously, their impact on prognosis will be changed. Through subgroup analysis, different miRNA panels led to different prognoses in patients with SCLC. In addition, there may be no significant difference in the length of follow-up (Fig. 9). This may be due to the high degree of malignancy and rapid progression of SCLC; most SCLC patients are in an advanced stage when they are examined, and their survival time is relatively short, which highlights the value of our study.

This meta-analysis is the first to study the impact of oncogenic and tumor suppressor microRNAs on the prognosis of patients with SCLC. Our goal is to obtain reliable biomarkers and provide valuable information for clinicians to treat SCLC patients effectively and adjust treatment strategies. For the discovery of tumor suppressor microRNAs, the function of miRNA can be enhanced through miRNA replacement therapy—that is, miRNA mimics can be used to enhance the antitumor effect of antitumor drugs [33]. The use of miRNA mimics may improve the expression of tumor suppressor microRNAs, delay the progression of malignancy, and improve the prognosis of SCLC patients. It has been found that oncogenic microRNAs can easily inhibit the activity of miRNAs and that the expression of oncogenic microRNAs can be reduced via miRNA antisense treatment and the use of miRNA inhibitors and oligomers, including RNA, DNA and DNA analogs, small molecule inhibitors, and miRNA sponges [33]. When oncogenic and tumor suppressor microRNAs act simultaneously, the combination of alternative therapy and antisense therapy can be used to improve the prognosis of SCLC patients.

Although our analysis shows that microRNAs play an important role in predicting the final outcome of SCLC patients, this study still has some limitations. First, due to the small number of articles and research subjects included in this meta-analysis, additional studies are necessary to confirm the prognostic value of oncogenic and tumor suppressor microRNAs in patients with SCLC (pooled HR was 1.60 (95% CI: 1.35–1.90; 0.42 (95% CI: 0.30–0.57)). Second, in the included studies, miRNA detection may affect the results of survival analysis. Third, this study shows that there is obvious heterogeneity in the demographic methods for determining SCLC and the measurement and adjustment of confounding factors. Although appropriate meta-analysis techniques are used for the random effects model, we cannot explain this difference. However, the sensitivity analysis found that the risk assessment is reliable in various quality factors. Fourth, some HRs could not be obtained directly from the included studies. We used Engauge Digitizer 11.1 to estimate the HRs based on Kaplan–Meier curves, which may reduce the reliability of our results.

## Conclusion

In conclusion, this meta-analysis demonstrated the role of microRNAs in predicting the prognosis of patients with SCLC. An increase in tumor-suppressive microRNAs and a decrease in oncogenic microRNAs are conducive to the overall survival rate of patients with advanced SCLC. This finding is useful for clinical practice. When oncogenic microRNAs are found in tissues, patients with SCLC need more urgent treatment. In addition, more research is needed to further identify tumor suppressor biomarkers in patients with lung cancer.

## Supplementary Information

**Additional file 1 **: **Supplementary Table 1**. The literature search strategy of PubMed.

## Data Availability

The datasets supporting the conclusions of this article are included within the article.

## Abbreviations

SCLC: Small cell lung cancer
NSCLC: Non-small cell lung cancer
HR: Hazard ratio
CI: Confidence interval
3′UTR: 3′ untranslated region
OS: Overall survival
PFS: Progression-free survival
NOS: Newcastle–Ottawa quality assessment scale
PKR: Protein Kinase RNA-activated
f-u time: Follow-up time

## References

[1] Siegel RL, Miller KD, Fuchs HE, Jemal A (2021). Cancer Statistics, 2021. CA Cancer J Clin.

[2] Ettinger DS, Wood DE, Aisner DL, Akerley W, Bauman J, Chirieac LR (2017). Non-small cell lung cancer, Version 5.2017, NCCN Clinical Practice Guidelines in Oncology. J Natl Compr Canc Netw.

[3] Wang S, Zimmermann S, Parikh K, Mansfield AS, Adjei AA (2019). Current diagnosis and management of small-cell lung cancer. Mayo Clin Proc.

[4] Rudin CM, Awad MM, Navarro A, Gottfried M, Peters S, Csoszi T (2020). Pembrolizumab or placebo plus etoposide and platinum as first-line therapy for extensive-stage small-cell lung cancer: randomized, double-blind, phase III KEYNOTE-604 study. J Clin Oncol.

[5] Sabari JK, Lok BH, Laird JH, Poirier JT, Rudin CM (2017). Unravelling the biology of SCLC: implications for therapy. Nat Rev Clin Oncol.

[6] Mancuso G, Bovio E, Rena O, Rrapaj E, Mercalli F, Veggiani C (2016). Prognostic impact of a 3-MicroRNA signature in cytological samples of small cell lung cancer. Cancer Cytopathol.

[7] Cao J, Song Y, Bi N, Shen J, Liu W, Fan J (2013). DNA methylation-mediated repression of miR-886-3p predicts poor outcome of human small cell lung cancer. Cancer Res.

[8] Zhou R, Zhou X, Yin Z, Guo J, Hu T, Jiang S (2015). Tumor invasion and metastasis regulated by microRNA-184 and microRNA-574-5p in small-cell lung cancer. Oncotarget.

[9] Liu H, Wu X, Huang J, Peng J, Guo L (2015). miR-7 modulates chemoresistance of small cell lung cancer by repressing MRP1/ABCC1. Int J Exp Pathol.

[10] Li M, Shan W, Hong B, Zou J, Li H, Han D (2020). Circulating miR-92b and miR-375 for monitoring the chemoresistance and prognosis of small cell lung cancer. Sci Rep.

[11] Bi N, Cao J, Song Y, Shen J, Liu W, Fan J (2014). A microRNA signature predicts survival in early stage small-cell lung cancer treated with surgery and adjuvant chemotherapy. PLoS One.

[12] Ranade AR, Cherba D, Sridhar S, Richardson P, Webb C, Paripati A (2010). MicroRNA 92a-2*: a biomarker predictive for chemoresistance and prognostic for survival in patients with small cell lung cancer. J Thorac Oncol.

[13] Moskwa P, Buffa FM, Pan Y, Panchakshari R, Gottipati P, Muschel RJ (2011). MiR-182-mediated downregulation of BRCA1 impacts DNA repair and sensitivity to PARP inhibitors. Mol Cell.

[14] Lai J, Yang H, Zhu Y, Ruan M, Huang Y, Zhang Q (2019). MiR-7-5p-mediated downregulation of PARP1 impacts DNA homologous recombination repair and resistance to doxorubicin in small cell lung cancer. BMC Cancer.

[15] Guo L, Liu Y, Bai Y, Sun Y, Xiao F, Guo Y (2010). Gene expression profiling of drug-resistant small cell lung cancer cells by combining microRNA and cDNA expression analysis. Eur J Cancer.

[16] Pan B, Chen Y, Song H, Xu Y, Wang R, Chen L (2015). Mir-24-3p downregulation contributes to VP16-DDP resistance in small-cell lung cancer by targeting ATG4A. Oncotarget.

[17] Ali Syeda Z, Langden SSS, Munkhzul C, Lee M, Song SJ (2020). Regulatory mechanism of microRNA expression in cancer. Int J Mol Sci.

[18] Sato J, Shimomura A, Kawauchi J, Matsuzaki J, Yamamoto Y, Takizawa S (2019). Brain metastasis-related microRNAs in patients with advanced breast cancer. PLoS One.

[19] Dawidowska M, Jaksik R, Drobna M, Szarzynska-Zawadzka B, Kosmalska M, Sedek L (2019). Comprehensive investigation of miRNome identifies novel candidate miRNA-mRNA interactions implicated in T-cell acute lymphoblastic leukemia. Neoplasia.

[20] Pavel AB, Campbell JD, Liu G, Elashoff D, Dubinett S, Smith K (2017). Alterations in bronchial airway miRNA expression for lung cancer detection. Cancer Prev Res (Phila).

[21] Weidle UH, Nopora A (2021). MicroRNAs involved in small-cell lung cancer as possible agents for treatment and identification of new targets. Cancer Genomics Proteomics.

[22] Feng S, Cong S, Zhang X, Bao X, Wang W, Li H (2011). MicroRNA-192 targeting retinoblastoma 1 inhibits cell proliferation and induces cell apoptosis in lung cancer cells. Nucleic Acids Res.

[23] Liao C, Chen W, Fan X, Jiang X, Qiu L, Chen C (2013). MicroRNA-200c inhibits apoptosis in pituitary adenoma cells by targeting the PTEN/Akt signaling pathway. Oncol Res.

[24] Li Q, Li X, Guo Z, Xu F, Xia J, Liu Z (2012). MicroRNA-574-5p was pivotal for TLR9 signaling enhanced tumor progression via down-regulating checkpoint suppressor 1 in human lung cancer. PLoS One.

[25] Gong L, Ren M, Lv Z, Yang Y, Wang Z (2018). miR-92b-3p promotes colorectal carcinoma cell proliferation, invasion, and migration by inhibiting FBXW7 in vitro and in vivo. DNA Cell Biol.

[26] Li C, Huo B, Wang Y, Cheng C (2019). Downregulation of microRNA-92b-3p suppresses proliferation, migration, and invasion of gastric cancer SGC-7901 cells by targeting Homeobox D10. J Cell Biochem.

[27] Wang Y, Lieberman R, Pan J, Zhang Q, Du M, Zhang P (2016). miR-375 induces docetaxel resistance in prostate cancer by targeting SEC23A and YAP1. Mol Cancer.

[28] Liu SL, Sui YF, Lin MZ (2016). MiR-375 is epigenetically downregulated due to promoter methylation and modulates multi-drug resistance in breast cancer cells via targeting YBX1. Eur Rev Med Pharmacol Sci.

[29] Lee K, Kunkeaw N, Jeon SH, Lee I, Johnson BH, Kang GY (2011). Precursor miR-886, a novel noncoding RNA repressed in cancer, associates with PKR and modulates its activity. RNA.

[30] Xiong S, Zheng Y, Jiang P, Liu R, Liu X, Chu Y (2011). MicroRNA-7 inhibits the growth of human non-small cell lung cancer A549 cells through targeting BCL-2. Int J Biol Sci.

[31] Zhen Y, Liu Z, Yang H, Yu X, Wu Q, Hua S (2013). Tumor suppressor PDCD4 modulates miR-184-mediated direct suppression of C-MYC and BCL2 blocking cell growth and survival in nasopharyngeal carcinoma. Cell Death Dis.

[32] Guo CM, Liu SQ, Sun MZ (2020). miR-429 as biomarker for diagnosis, treatment and prognosis of cancers and its potential action mechanisms: a systematic literature review. Neoplasma.

[33] Gambari R, Brognara E, Spandidos DA, Fabbri E (2016). Targeting oncomiRNAs and mimicking tumor suppressor miRNAs: new trends in the development of miRNA therapeutic strategies in oncology. Int J Oncol.

