# Supplementary material for: Prognostic value of microRNAs in patients with small cell lung cancer: a meta-analysis
Source: World J Surg Oncol. 2022 Dec 4;20:381. doi: 10.1186/s12957-022-02851-4 (PMC9720993; doi:10.1186/s12957-022-02851-4)
Supplement: Supplementary file 1 — Additional file 1 : Supplementary Table 1. The literature search strategy of PubMed. [file 12957_2022_2851_MOESM1_ESM.docx]

the literature search strategy of PubMed

#1 search" small cell lung cancer "[MeSH]

#2 search" small cell lung cancer "

#3 search" SCLC "

#4 #1 OR #2 OR #3

#5 search " microRNAs "[MeSH]

#6 search " microRNAs "

#7 search " microRNA"

#8 search " miRNAs "

#9 search " miRNA"

#10 search " miR"

#11 #5 OR #6 OR #7 OR #8 OR #9 OR #10

#12 search" prognosis "[MeSH]

#13 search" prognosis "

#14 search" survival "

#15 #12 OR #13 OR #14

#16 #4 AND #11 AND #15

the literature search strategy of Embase

'small cell lung cancer'/exp AND 'microrna'/exp AND 'prognosis'/exp NOT 'non small cell lung cancer'/exp

the literature search strategy of Web of Science

(((ALL=(small cell lung cancer )) AND ALL=(microRNAs)) AND ALL=(prognosis )) NOT ALL=(non small cell lung cancer )
